# Efficiency of newly formulated camptothecin with β-cyclodextrin-EDTA-Fe_3_O_4_ nanoparticle-conjugated nanocarriers as an anti-colon cancer (HT29) drug

**DOI:** 10.1038/s41598-017-09140-1

**Published:** 2017-09-08

**Authors:** Poorani Krishnan, Mariappan Rajan, Sharmilah Kumari, S. Sakinah, Sivan Padma Priya, Fatin Amira, Lawal Danjuma, Mok Pooi Ling, Sharida Fakurazi, Palanisamy Arulselvan, Akon Higuchi, Ramitha Arumugam, Abdullah A. Alarfaj, Murugan A. Munusamy, Rukman Awang Hamat, Giovanni Benelli, Kadarkarai Murugan, S. Suresh Kumar

**Affiliations:** 1 Department of Medical Microbiology and Parasitology, Universiti Putra Malaysia, 43400 UPM Serdang Selangor, Malaysia; 2 Department of Natural Products Chemistry, School of Chemistry, Madurai Kamaraj University, Madurai, 625 021 Tamil Nadu India; 3 Department of Biomedical Science, Universiti Putra Malaysia, 43400 UPM Serdang, Selangor Malaysia; 4 Genetics and Regenerative Medicine Research Centre, Universiti Putra Malaysia, 43400 UPM Serdang, Selangor Malaysia; 5 Laboratory of Vaccines and Immunotherapeutic, Institute of Bioscience, Universiti Putra Malaysia, 43400 UPM Serdang Selangor, Malaysia; 6 Department of Chemical and Materials Engineering, National Central University, Jhong-li, Taoyuan, 32001 Taiwan; 7 Department of Reproduction, National Research Institute for Child Health and Development, Tokyo, 157-8535 Japan; 8 Department of Botany and Microbiology, King Saud University, Riyadh, 11451 Saudi Arabia; 9 Department of Biology, Faculty of Science, Universiti Putra Malaysia, 43400 UPM Serdang, Selangor Malaysia; 10 Department of Agriculture, Food and Environment, University of Pisa, via del Borghetto 80, 56124 Pisa, Italy; 11 Division of Entomology, Department of Zoology, School of Life Sciences, Bharathiar University, Coimbatore, Tamil Nadu India; 12 Muthayammal Centre for Advanced Research, Muthayammal College of Arts and Science, Rasipuram, Namakkal, Tamilnadu 637408 India; 13 The BioRobotics Institute, Scuola Superiore Sant’Anna, viale Rinaldo Piaggio 34, 56025 Pontedera, Pisa Italy

## Abstract

Camptothecin (CPT) is an anti-cancer drug that effectively treats various cancers, including colon cancer. However, poor solubility and other drawbacks have restricted its chemotherapeutic potential. To overcome these restrictions, CPT was encapsulated in CEF (cyclodextrin-EDTA-FE_3_O_4_), a composite nanoparticle of magnetic iron oxide (Fe_3_O_4_), and β-cyclodextrin was cross-linked with ethylenediaminetetraacetic acid (EDTA). This formulation improved CPT’s solubility and bioavailability for cancer cells. The use of magnetically responsive anti-cancer formulation is highly advantageous in cancer chemotherapy. The chemical characterisation of CPT-CEF was studied here. The ability of this nano-compound to induce apoptosis in HT29 colon cancer cells and A549 lung cancer cells was evaluated. The dose-dependent cytotoxicity of CPT-CEF was shown using MTT. Propidium iodide and Annexin V staining, mitochondrial membrane depolarisation (JC-1 dye), and caspase-3 activity were assayed to detect apoptosis in CPT-CEF-treated cancer cells. Cell cycle analysis also showed G1 phase arrest, which indicated possible synergistic effects of the nano-carrier. These study results show that CPT-CEF causes a dose-dependent cell viability reduction in HT29 and A549 cells and induces apoptosis in colon cancer cells via caspase-3 activation. These data strongly suggest that CPT could be used as a major nanocarrier for CPT to effectively treat colon cancer.

## Introduction

The use of magnetic nanoparticles (MNPs) in the field of biomedical applications, such as magnetic drug delivery, magnetic resonance imaging, transfection, and cell and tissue targeting, has drawn considerable attention owing to their intrinsic magnetic properties^1^. MNPs show superparamagnetic behaviour, which permits them to gain magnetism in an applied magnetic field and lose it when the field is removed^2^. This property of MNPs is fully realised when they are used as drug delivery agents, whereby chemotherapeutic drugs can be targeted to desired locations in the body by application of an external magnetic field. The combination of MNPs and external magnetic field provides two unique advantages that benefit medicine immensely^3^. Priyanka Sharma *et al*. synthesized a biocompatible, water-dispersible phosphate affixed iron oxide magnetic drug vehicle by a superficial chemical method for anti-cancer drug delivery^4^.

Polymers not only have a considerable potential for drug delivery^5^, but also can be used for medical devices, wound dressing, and fabricating scaffolds in tissue engineering^6^. Cyclodextrins are candidates for such a role, because of their ability to alter the physical, chemical, and biological properties of guest molecules through the formation of inclusion complexes. Recently, various cyclodextrin derivatives have been prepared to extend the physicochemical properties and inclusion capacity of cyclodextrin as novel drug carriers. Camptothecin (CPT) is a major anti-cancer drug that shows efficacy toward many cancers, including ovarian and colorectal tumours. CPT is an alkaloid isolated in the early 1960s from the Chinese tree, *Camptotheca acuminata* ^7^. CPT is a selective topoisomerase I inhibitor^8^. CPT’s ability to inhibit nitric oxide (NO) biosynthesis has also been proposed to contribute to its anti-tumour activity^9^. As a DNA topoisomerase I inhibitor, CPT forms a stable, ternary topoisomerase I-DNA cleavable complex, which initiates an apoptotic signalling pathway, ultimately resulting in cell death^10^. However, a major drawback of CPT is its reduced therapeutic potential owing to 1) poor solubility in aqueous media^11^ and 2) active lactone ring instability at physiological pH^12^. Given that chemotherapy is a widely used cancer treatment, various nanocarriers are continuously being formulated and designed to enhance the solubility of chemotherapeutic drugs such as CPT. The solubility of chemotherapy drugs is critical because it affects delivery and bioavailability at the targeted location. Solubility limitations have greatly reduced the ability of chemotherapy drugs, such as CPT, to exert their anti-cancer properties, which limits their use for only a subset of cancers. To overcome this problem, multiple analogues of CPT have been developed with improved lactone stability and aqueous solubility. Various polymeric conjugates of CPT, including polyethylene glycol (PEG)^11^, cyclodextrin copolymer^13^, poly (L-glutamic acid)^14^, and chitosan^15^, have been investigated previously. Studies are ongoing in order to synthesis effective, water-soluble analogues of CPT to enhance its anti-cancer potential. With this study objective, we conjugated CPT with β-cyclodextrin and iron NPs (Fe_3_0_4_) and cross-linked using EDTA to achieve a soluble CPT analogue (CPT-CEF) that was designed to improve the efficiency of CPT as an anti-cancer drug. We then tested the ability of CPT-CEF to induce apoptosis in the human colon adenocarcinoma cell line, HT29. Additionally, the drug was also concurrently tested on A549 lung cancer cells to reflect on the drug ability to be used in other cancers besides colon cancer. In this study, we provide further insight into the potential of this water-soluble formulation to enhance the anti-tumour activity of pure CPT. To the best of our knowledge, the functionalization of iron NPs on β-CD was carried with simple and sustainable method and the combination of CEF as a nanocarrier for CPT is being studied for the first time as an effective nanocarrier.

## Results

### FT-IR analysis

**Figure 1:**
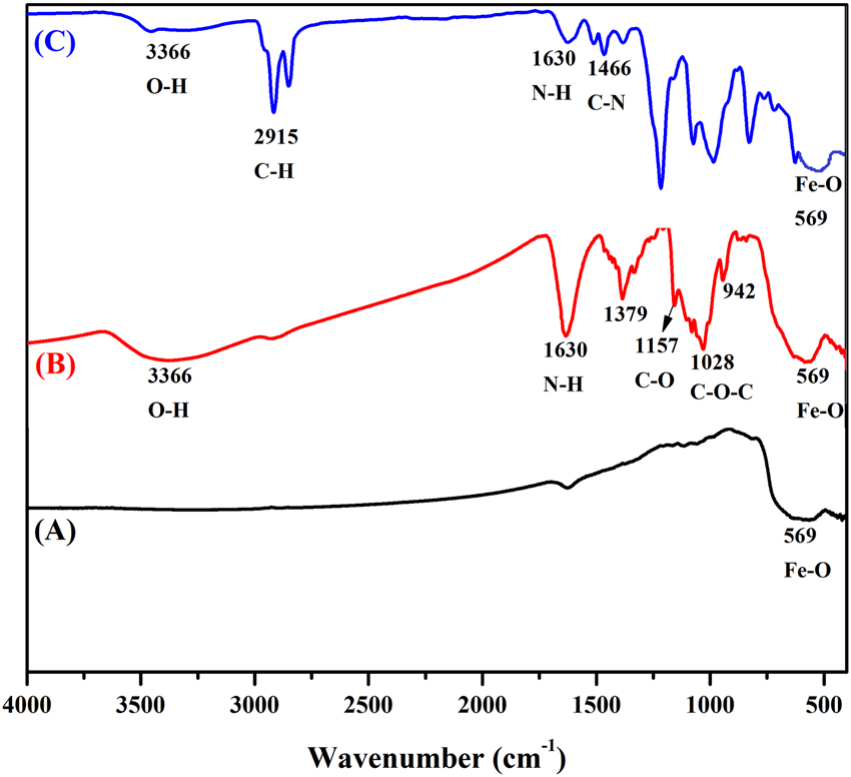
FT-IR spectra for (**A**) Fe_3_O_4_, (**B**) β-CD-EDTA-Fe_3_O_4_, and (**C**) β-CD-EDTA-Fe_3_O_4_/CPT nanocarriers.

FT-IR analysis was performed to confirm the formation of Fe_3_O_4_ NPs, formation of β-CD-EDTA-Fe_3_O_4_ nanocarriers, and encapsulation of the CPT drug on the β-CD-EDTA-Fe_3_O_4_ nanocarriers. FT-IR spectra of Fe_3_O_4_ NPs, β-CD-EDTA-Fe_3_O_4_ nanocarriers, and CPT-loaded β-CD-EDTA-Fe_3_O_4_ nanocarriers are shown in Fig. 1(A,B,C) respectively. The peaks appeared at 569 cm^−1^, characteristic vibration of the Fe-O bond, indicating the formation of Fe_3_O_4_ NPs (Fig. 1A). This confirms the presence of the magnetic core, and thus is more pronounced in the bare magnetite NPs^16^. The spectrum of β-CD-EDTA nanocarriers showed the characteristic peaks of β-CD at 942, 1,028, 1,157, and 1,630 cm^−1^ (Fig. 1B). The peak at 942 cm^−1^ was due to the R-1, 4-bond skeleton vibration of β-CD, the peak at 1,028 cm^−1^ corresponded to the anti-symmetric glycosidic ν_a_ (C–O–C) vibration, the peak at 1,197 cm^−1^ was due to the coupled ν(C–C/C–O) stretch vibration, and the peak at 1,630 cm^−1^ corresponded to N-H bending vibrations. A broad spectrum around 3,366 cm^−1^ was also observed, which was assigned to the hydroxyl group of the β-CD. These peaks indicate that β-CD-EDTA had been successfully combined with Fe_3_O_4_ NPs. The encapsulation of the CPT drug in β-CD**-**EDTA-Fe was confirmed by FT-IR spectrum (Fig. 1C), which shows peaks at 1,075 and 2,915 cm^−1^ corresponding to C-O and C-H stretching vibrations in the drug. The hydroxyl stretching vibration of CPT at about 3,430 cm^−1^ and that of β-CD**-**EDTA-Fe at 3,336 cm^−1^ disappeared in the FT-IR spectrum because of the formation of hydrogen bonds. These data show that CPT had been successfully loaded with the β-CD-EDTA-Fe_3_O_4_ nanocarrier.

### Morphological analysis by transmission electron microscopy (TEM)

**Figure 2:**
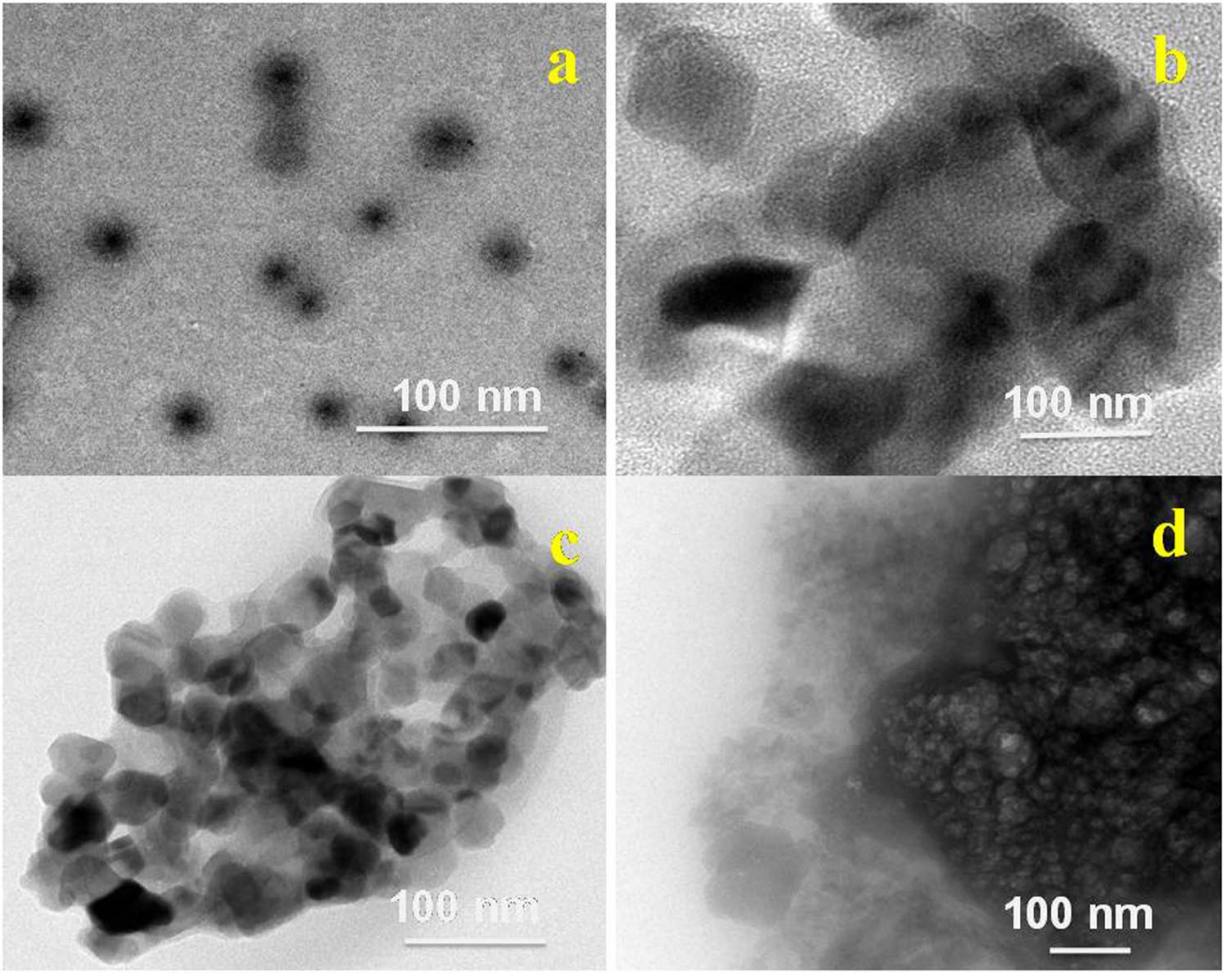
TEM of (**a**) Fe_3_O_4;_ (**b**) β-CD-EDTA; (**c**) β-CD-EDTA-Fe_3_O_4_; (**d**) β-CD-EDTA-Fe_3_O_4_/CPT Nanocarriers.

The morphology of the prepared Fe-MN particles, β-CD-EDTA, β-CD-EDTA-Fe_3_O_4,_ and β-CD-EDTA-Fe_3_O_4_/CPT,  was investigated using TEM. The formed Fe NPs (Fig. 2a) exhibited a spherical shape, smooth surface, and uniform arrangement. The modified β-CD with EDTA had dispersed spherical morphology and some vast and unpredictably shaped aggregates were obtained after cross linking of EDTA on CD (β-CD-EDTA, Fig. 2b), and combining the Fe NPs with the β-CD-EDTA carriers produced structures with a smooth surface and good incorporation (β-CD-EDTA-Fe_3_O_4,_ Fig. 2c). When CPT was encapsulated within the β-CD-EDTA-Fe_3_O_4_ carriers, the resulting β-CD-EDTA-Fe_3_O_4_/CPT inclusion complexes drastically changed shape and morphology, becoming more amorphous (Fig. 2d). The change in the surface morphology of the inclusion complexes was indicative of the presence of a new solid phase, which might be due to the molecular encapsulation of CPT into β-CD-EDTA-Fe_3_O_4_. TEM images confirmed that the individual components of the magnetic nanocarrier (β-CD-EDTA-Fe_3_O_4_) and drug loaded magnetic carrier (β-CD-EDTA-Fe_3_O_4_/CPT), had nanometer size range with a separated particles. After the incorporation was performed, distinct drug particles with a dense structure were observed (Fig. 2d).

### Particle size analysis

Synthesised Fe_3_O_4_, β-CD-EDTA, β-CD-EDTA-Fe_3_O_4_, and β-CD-EDTA-Fe_3_O_4_/CPT nanocarriers were analysed for size and polydispersity index by a particle size analyser. The results are shown the Table 1, and the instrumental data are shown in Supplementary Figures S1–S4. The average particle sizes of the Fe_3_O_4_, β-CD-EDTA, β-CD-EDTA-Fe_3_O_4,_ and β-CD-EDTA-Fe_3_O_4_/CPT NPs are 168, 338, 308, and 386 nm, respectively. Fe MNs conjugation of with EDTA cross linked β-CD formed a carrier with more compact structure. Subsequent addition of CPT anticancer drug on the β-CD-EDTA-Fe_3_O_4_ carriers increases their size. These results correlated well with the TEM results of Fig. 2. In the TEM, the surface of β-CD-EDTA-Fe_3_O_4_ possesses cavity-like morphology, which is filled by the addition of the CPT drug, increasing the particle size. The polydispersity index decreases with increasing of particle size, it demonstrate increasing stability of the particles. From the results, β-CD-EDTA-Fe_3_O_4_ appears to be more stable (Table 1).

**Table 1:** Particle size and polydispersity index analyses of Fe_3_O_4_, β-CD-EDTA, β-CD-EDTA-Fe_3_O_4,_ and β-CD-EDTA-Fe_3_O_4_/CPT.

Name of the sample	Particle size (nm)	Polydispersity index
Fe_3_O_4_	168	0.331
β-CD-EDTA	338	0.256
β-CD-EDTA-Fe_3_O_4_	308	0.227
β-CD-EDTA-Fe_3_O_4_/CPT	386	0.291

### Zeta potential measurements

The zeta potential is an important marker of the stability of various colloidal dispersions. The values of the zeta potential significantly showed the extent of electrostatic repulsion between all the adjacent, like charged particles in a solution or dispersion. The zeta potential of the drug-unloaded β-CD-EDTA-Fe_3_O_4_ and drug-loaded β-CD-EDTA-Fe_3_O_4_ nanocarriers was evaluated to estimate the colloidal stability of the NPs. Zeta potential is an important index for the stability of β-CD-EDTA-Fe_3_O_4_ and β-CD-EDTA-Fe_3_O_4_/CPT nanocarriers, as shown in Figures S5 and S6, respectively. A slight decrease from −2.46 to −2.95 mV was observed in the zeta potential value of β-CD-EDTA-Fe_3_O_4_/CPT (Figure S6) and β-CD-EDTA-Fe_3_O_4_ (Figure S5). In the case of the encapsulation with CPT, the significantly changed colloidal stability of the nanoparticles and it indicates the success of drug loading on the carrier. As per the most widely accepted DLVO (named after inventors Derjaguin, Landau, Verwey and Overbeek) theory colloid stability depends on the sum of van der Waals attractive forces and electrostatic repulsive forces due to the electric double layer. This decrease of zeta potential is likely caused by the addition of carboxyl groups from the CPT drug molecules. It confirms loading CPT drug on the carrier as well as the CPT loaded carrier more stable.

### *In-vitro* drug release studies

**Figure 3:**
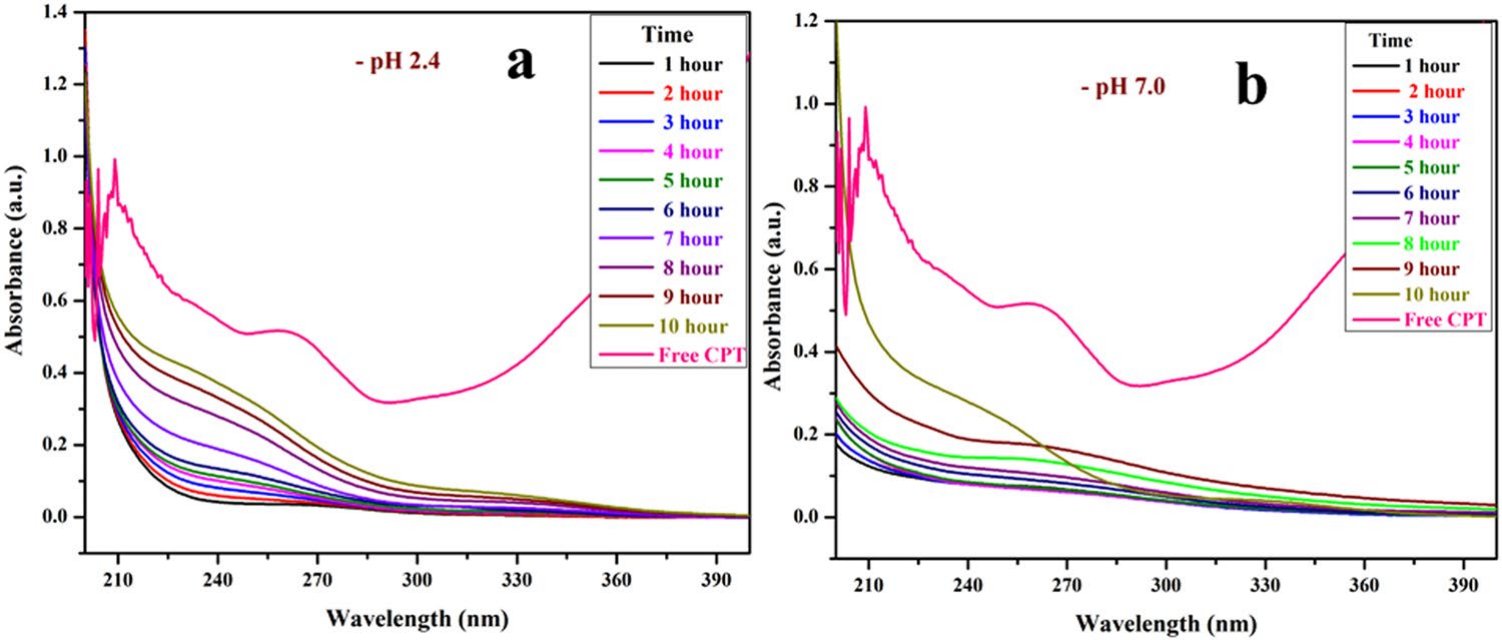
*In-vitro* drug release analysis of β-CD-EDTA-Fe_3_O_4_/CPT at pH 2.4 (**a**) and at pH 7.0 (**b**).

Drug release studies are conducted to study the rate at which the loaded drug is released into the environment. Drug release studies are performed at biologically relevant pH and temperatures. *In-vitro* CPT drug release profile from β-CD-EDTA-Fe_3_O_4_ carriers were assessed using the dialysis technique at pH 2.4 and pH 7.0 at 37 °C. As shown in (Fig. 3) nearly 65% and 58% of CPT was released within 10 hours at pH 2.4 and 7.0 respectively. At pH 7.0, the release of CPT is about 58% over a period of 10 hours, indicating that β-CD-EDTA-Fe_3_O_4_-CPT nano-carriers remain stable in the physiological condition. When pH is changed to 2.4 CPT is released more rapidly from the β-CD-EDTA-Fe_3_O_4_/CPT nanocarriers than pH 7.0. When treated in acidic condition at pH 2.4 conditions, the release rate is remarkably promoted. These results are consistent with the fact that CPT degrades much more quickly with acidic condition. The absorbance value increased with respect to the time CPT drug released from the carrier. From this study we confirm the drug was successfully released from the β-CD-EDTA-Fe_3_O_4_ carrier at pH 2.4 and pH 7.0. The UV absorption peak is shifted to shorter wavelengths with an increase in the concentration of drug and dilution of the carriers, accompanied by the increase in absorbance. Similar behaviors of CD with various drugs by UV–visible spectroscopy have been reported in literature^17, 18^.

### Magnetic properties studies

**Figure 4:**
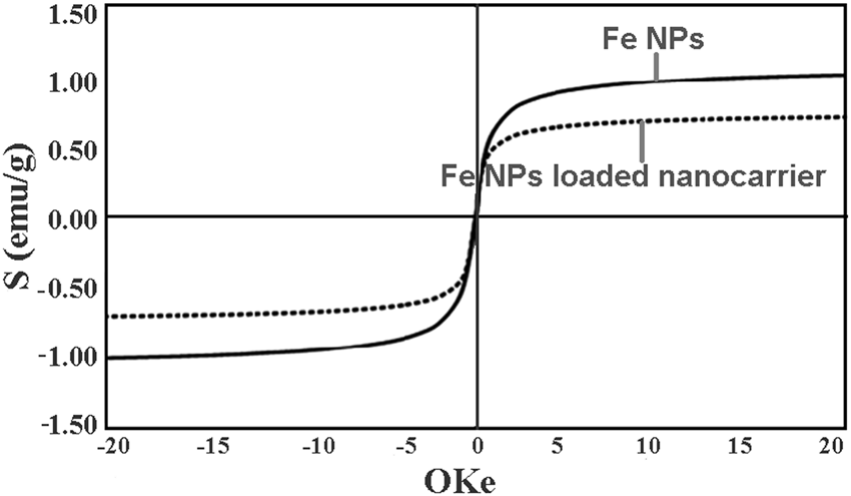
Magnetic properties of CPT-CEF determined through magnetometer.

Magnetic properties of the iron nanoparticles and iron nanoparticles loaded nanocarriers (CEF) was tested in vibrating sample magnetometer (VSM, Dexing, Model: 250) with a sensitivity of 50 emu. From this study, we observed that the magnetic properties of the Fe were retained after its functionalization in the nanocarriers (Fig. 4). This data is essential in reflecting the magnetic properties of CPT-CEF thus suggesting its potential to be utilized in magnetically targeted cancer therapy.

### The effect of CPT-CEF on HT29 and A549 cell viability

**Figure 5:**
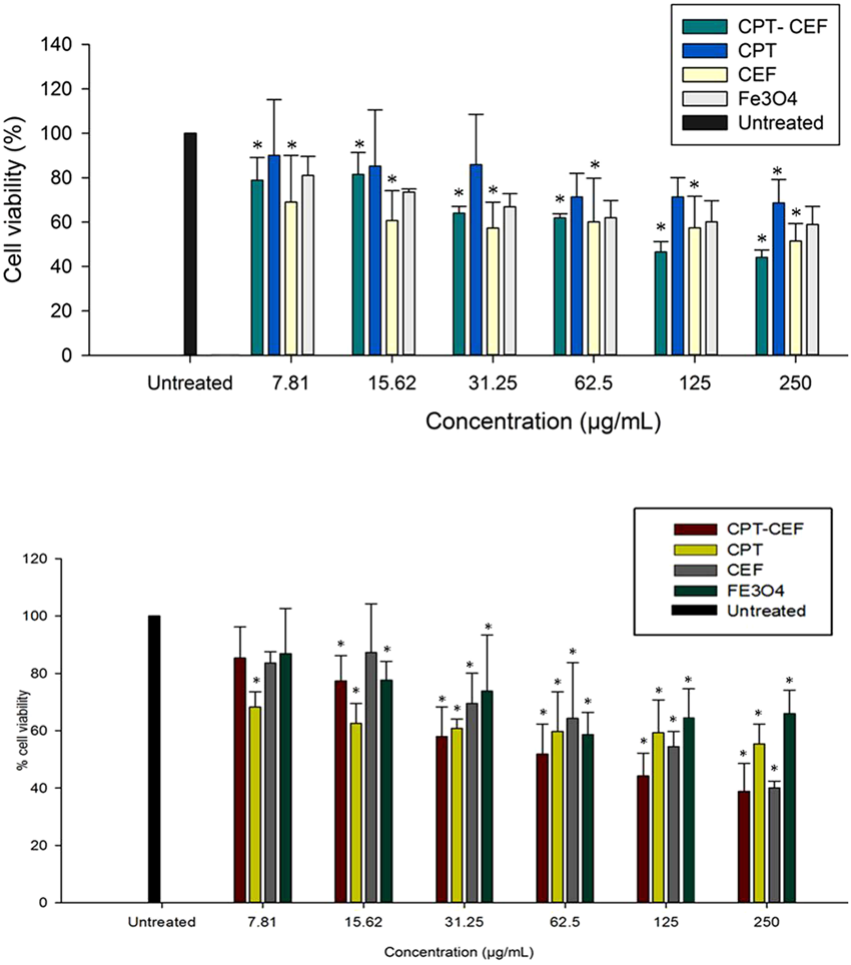
(**a**) The effect of CPT-CEF on cell viability of HT29 colon cancer cells and (**b**) The effect of CPT-CEF on cell viability of A549 lung cancer cells. Cells were treated with various concentrations of CPT-CEF, CPT, CEF and Fe_3_O_4_ for 48 h, and cell viability was assessed via MTT assay. CPT-CEF reduced cell viability in HT29 cells in a dose-dependent manner. Results (mean ± SD) were calculated as percent of corresponding control values. *P < 0.05 is significant. Statistical analysis was performed via ANOVA. Each point represents three independent experiments.

To determine the effect of CPT-CEF on the viability of HT29 colon cancer cells, an MTT assay was used. MTT assays are indicative of the impact of CPT-CEF on the mitochondrial activity of treated cancer cells, thus reflecting cell cytotoxicity. HT29 and A549 cells were treated with various concentrations of CPT-CEF, free CPT, free CEF, and Fe_3_O_4_ at three different time points of 24, 48, and 72 h. The MTT assay results (Fig. 5a and b) showed a concentration-dependent decrease in cell viability of HT29 and A549 cancer cells respectively when compared to untreated cells, thus indicating the ability of CPT-CEF to retain the anticancer activity of CPT. A significant cell viability decrease was observed at a CPT-CEF concentration of 100 µg/mL. The effective CPT-CEF concentration for 50% inhibition (IC_50_) of HT29 cell growth after 48 h was 133.5 μg/m (Fig. 5a). The IC_50_ concentration of CPT for treatment with HT29 was observed to be beyond a range of 250 μg/mL thus indicating the potential of CPT-CEF to provide significant impact on HT29 cancer cells at low concentration of loaded CPT. In addition, treatment with CEF alone was mildly cytotoxic to HT29 cells. Also significant cell viability decrease in CPT-CEF treated A549 cells were observed at a CPT-CEF concentration of 85 μg/mL making it the effective CPT-CEF concentration for achieving 50% inhibition (IC_50_) in A549 cell growth after 48 h of treatment (Fig. 5b). To evaluate further the capacity of CPT-CEF to induce apoptosis in cancer cell line, HT29 cell line model was selected as CPT is known to be utilized widely in colon cancer treatment.

### CPT-CEF causes morphological changes in HT29 cancer cells

**Figure 6:**
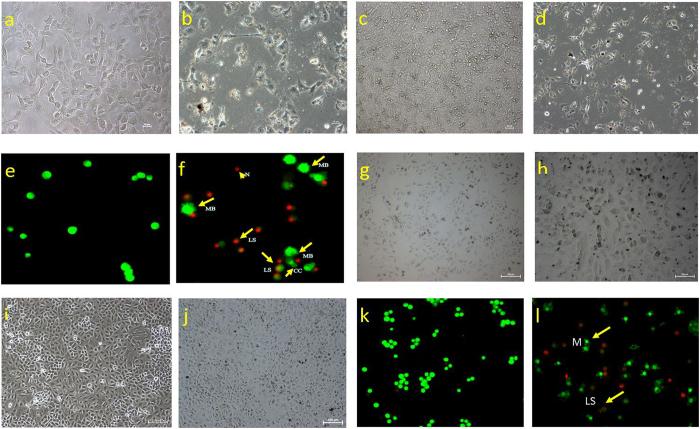
Microscopy of HT29 (**a,b,c,d,e,f**) and A549 (**g,h,i,j,k,l**) cells treated with CPT-CEF to show changes in cell morphology. Cells were exposed to the IC_50_ concentration of CPT-CEF and viewed at 24 and 48 h. Panel a and **g** (Untreated cells at 24 h), **b** and **h** (IC_50_ treated cells for 24 h), **c** and **i** (Untreated cells at 48 h), and d and j (IC_50_ treated cells for 48 h). Under phase contrast microscopy, cell morphology changes in treated cells are clearly visible in comparison to untreated cells; the cell membrane is evidently damaged. Panels’ e and f show fluorescent micrographs of AO/PI double-stained human colorectal cancer cells (HT29). Cells were exposed to the IC_50_ concentration of CPT-CEF (panel f and i) or vehicle (panel e and k) for 48 h. Cell apoptosis was assayed by AO/PI staining to detect chromosomal condensation (CC), late stage apoptosis (LS), necrosis (N), and membrane blebbing (MB), as shown in the micrograph. Microscope magnification × 100 and scale bar of 10 µm (HT29) and scale bar of 100 µm (A549) were applied for the images.

HT29 and A549 cells were treated with the IC_50_ concentration of CPT-CEF (derived from MTT assay) for and the morphological changes in the cells were observed under phase contrast microscopy. In Fig. 6.1, the changes in appearance of treated HT29 cells (Fig. 6b,d) compared to that of untreated cells (Fig. 6.1a,c) are shown. At 24 h, some cell damage was observed in treated cells, with ruptured membranes and altered nuclear morphology visible in some cells. After 48 h, control cells (Fig. 6.1c) still maintained intact cell membranes and minimal cell detachment. However, treated cells for 48 h (Fig. 6.1d) exhibits drastic difference in cells structure where a large proportion of cells lost cell membrane structure completely and nuclear fragmentation is clearly evident. Similar morphological changes were observed in CPT-CEF treated A549 cells (Fig. 6.2) indicating the potential of CPT-CEF to induce cell toxicity in various cancer cells.

### Membrane blebbing detected using AO/PI staining assay

Cell death can occur by apoptosis or necrosis. In this study, AO/PI double staining was used to determine the mode of death of HT29 and A549 cells treated with IC_50_ concentrations of CPT-CEF for 48 h derived from respective MTT assay. AO/PI double staining distinguishes viable, apoptotic, and necrotic cells. Acridine orange (AO) stains viable cells, while propidium iodide (PI) intercalates into and stains double-stranded DNA of dead cells that have lost plasma membrane integrity. Viable cells have round and green nuclei. The nuclei of cells undergoing apoptosis are also stained green, but the nuclei appear fragmented. Late apoptotic and necrotic cells appear orange and red, respectively^19^. From the data in Fig. 6 HT29 (f) and A549 (l) we conclude that CPT-CEF treatment for 48 h with the IC_50_ concentration of CPT-CEF causes characteristics of early apoptosis in both cell line, such as cell shrinkage (CS), plasma membrane blebbing (MB), and chromatin condensation (CC). Late stage apoptotic features are also detected (LA). The untreated cells (Fig. 6e and k) mostly stained green and remained intact. Based on the results of AO/PI staining we conclude that CPT-CEF, at a concentration of 133.5 μg/mL for HT29 and 85 μg/mL for A549 cancer cells, has a significant impact on the cell membrane and nuclear membrane of the respective cells.

### CPT-CEF induces apoptosis in HT29 and A549 cancer cells

**Figure 7:**
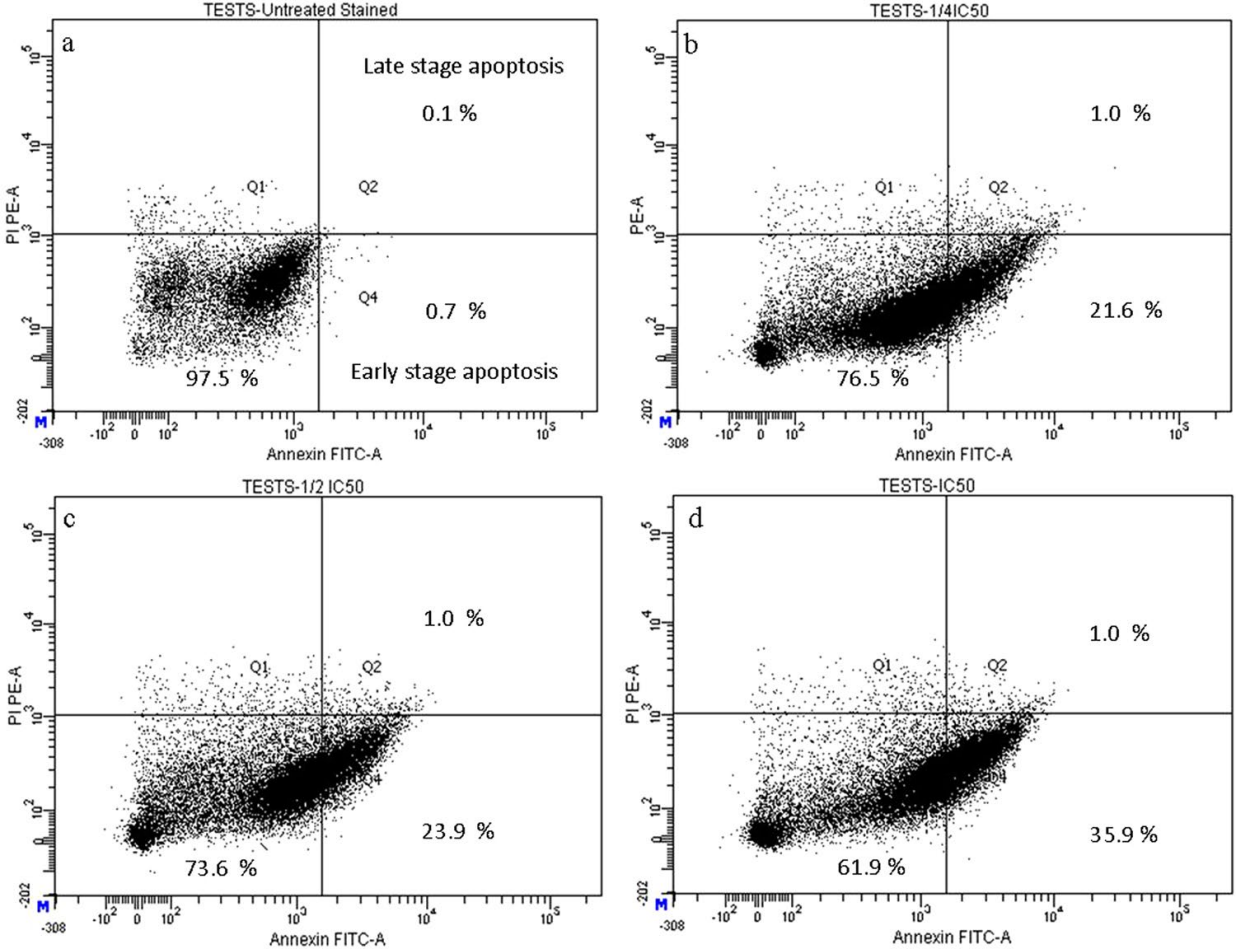
Annexin V/PI assay showing the apoptosis-inducing effect of CPT-CEF. HT29 cells were treated with various concentrations of CPT-CEF: ¼ IC_50_ (panel b), ½IC_50_ (panel c), IC_50_ (panel d), or untreated (panel a) for 48 h, and viability was assessed via Annexin V/PI assay and flow cytometry.

**Figure 8:**
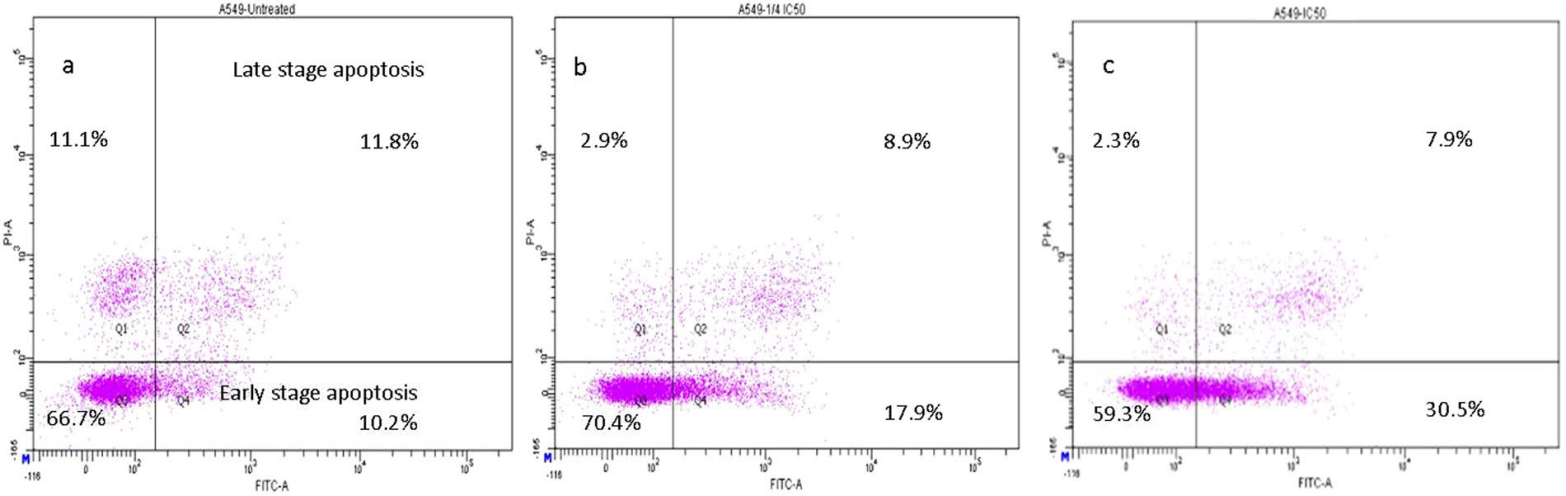
Annexin V/PI assay showing the apoptosis-inducing effect of CPT-CEF. A549 cells were treated with various concentrations of CPT-CEF: ¼ IC_50_ (panel b), IC_50_ (panel c), or untreated (panel a) for 48 h, and viability was assessed via Annexin V/PI assay and flow cytometry.

To evaluate whether the CPT-CEF-induced inhibition of cell proliferation was related to cell apoptosis, the effect of CPT-CEF on cell apoptosis was evaluated via Annexin V/PI staining. Cancer cells were exposed to either the IC_50_, ½ IC_50,_ or ¼ IC_50_ of CPT-CEF for 48 h, and analysed by flow cytometry using FITC-conjugated Annexin V (FL1-H) and PI (FL2-H) double staining (Fig. 7). The data show a significant increase in the percentage of both early (Annexin V positive, PI negative) and late (Annexin V positive, PI positive) apoptotic cells in a CPT-CEF concentration-dependent manner. The number of cells entering early stage apoptosis reached about 35.9% with IC_50_ treatment. These results suggest that CPT-CEF has retained the apoptosis-inducing potential of CPT in colon cancer cell lines. The data clearly show an increasing trend in early stage apoptosis with increasing concentration of CPT-CEF. The IC_50_ concentration of CPT-CEF significantly induced early apoptosis in treated HT29 cells compared to untreated cells. Similar results were seen in A549 treated cells whereby after 48 h of IC_50_ treatment with CPT-CEF, the number of cells in late stage apoptosis was about 30.5% (Fig. 8). These results suggested that CPT-CEF has the potential to induce apoptosis in lung cancer cell lines.

### CPT-CEF induces apoptosis in HT29 and A549 cancer cells by altering mitochondrial membrane potential

**Figure 9:**
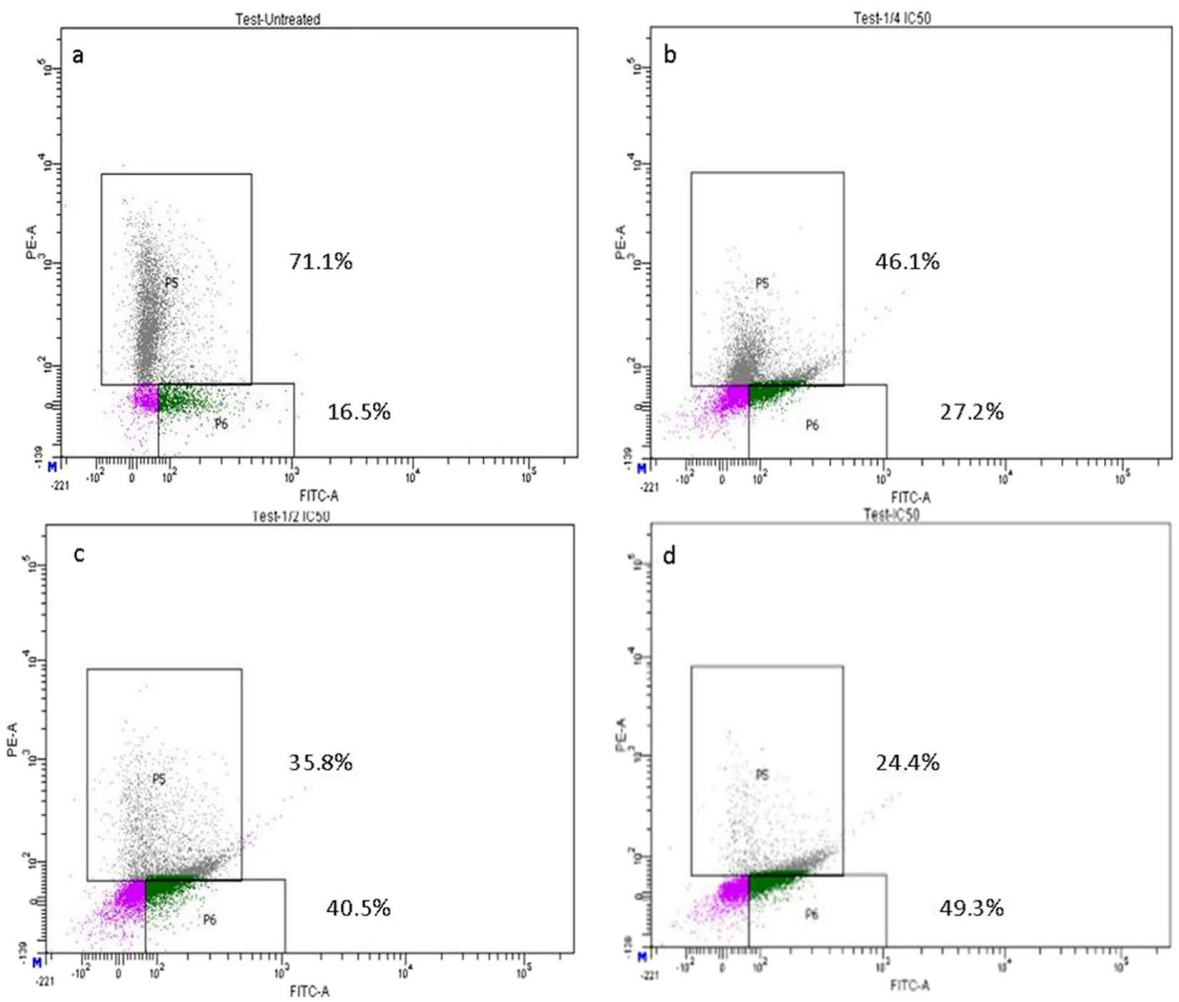
Mitochondrial depolarisation initiated by CPT-CEF. As analysed with JC-1 dye and flow cytometry, CPT-CEF treatments of HT29 cells with concentrations of ¼ IC_50_ (panel b), ½ IC_50_ (panel c), IC_50_ (panel d), or untreated (panel a) for 48 h are shown.

**Figure 10:**
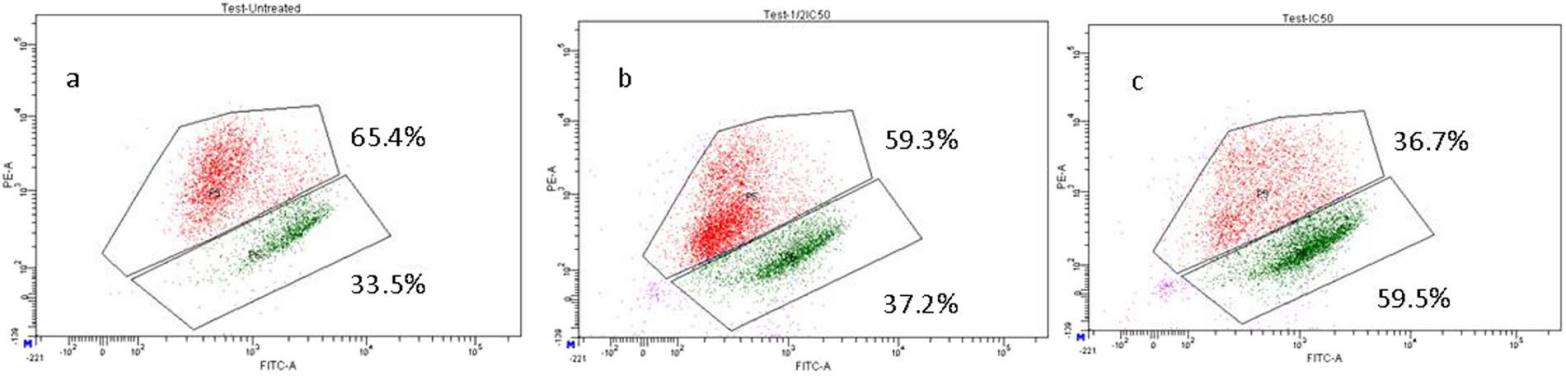
Mitochondrial depolarisation initiated by CPT-CEF. As analysed with JC-1 dye and flow cytometry, CPT-CEF treatments of A549 cells with concentrations of ½ IC_50_ (panel b), IC_50_ (panel c), or untreated (panel a) for 48 h are shown.

To evaluate whether CPT-CEF causes alterations to mitochondrial membrane potential (Δ*Ψ*_M_) of colon cancer cells, JC-1 dye was used. At higher potential, JC-1 aggregates in the mitochondria and fluoresces red; at lower potential, it loses its ability to form aggregates in the mitochondria, remains as a monomer in the cytoplasm, and fluoresces green^20^. In this study, HT29 cells were exposed to IC_50_, ½ IC_50,_ and ¼ IC_50_ concentrations of CPT-CEF for 48 h, and then were analysed via flow cytometry. The data (Fig. 9) show a significant increase in the percentage of mitochondrial membrane depolarised cells in a concentration-dependent manner. At the IC_50_ concentration, about 49% are observed to be Δ*Ψ*_M_ depolarised cells. Also, in A549 IC_50_ treated cells about 60%, of the treated cells were observed to have experienced changes (depolarizations) in Δ*Ψ*_M_ (Fig. 10). These results suggest that CPT-CEF has the ability to affect mitochondrial membrane potential of cancer cells. During apoptosis, cell membranes are damaged, thus causing an alteration in the Δ*Ψ*_M_. JC-1 staining shows that the IC_50_ concentration (Fig. 9) of CPT-CEF more than doubles the mitochondrial membrane depolarisation observed in untreated cells. These data suggest that CPT-CEF induces apoptosis, accompanied by alterations in the mitochondrial membrane potential.

### CPT-CEF induces caspase-3 protein expression in HT29 cancer cells

**Figure 11:**
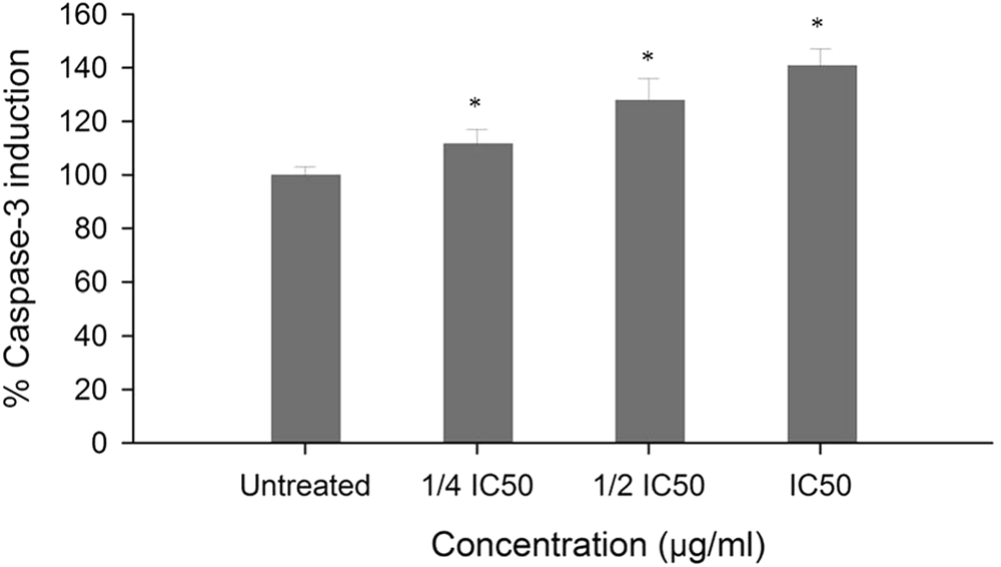
Caspase-3 induction in various CPT-CEF-treated HT29 cells. Graph shows the percentage of induction of caspase-3 activity in CPT-CEF-treated HT29 cells at a concentration of ¼ IC_50_, ½ IC_50_, IC_50_ and untreated for 48 h. Results are presented as mean ± S.D. (n = 3). *P < 0.05, compared with the control group (Untreated).

The release of cytochrome c from mitochondria into the cytosol leads to an activation of the caspase cascade, including caspase-3 activation^21^. Caspase-3, also known as CPP32/Yama/Apopain, is a key mediator of apoptosis^22^. To substantiate the presence of activated caspase-3 in CPT-CEF-treated cells, a colorimetric assay was performed for capsase-3, using its specific substrate poly (ADP-ribose) polymerase, containing the amino acid motif DEVD conjugated to p-nitroaniline (pNA). The role of caspase-3 in the CPT-CEF-induced apoptosis was investigated with IC_50_, ½ IC_50,_ and ¾ IC_50_ concentrations. As shown in Fig. 11, HT29 cells treated for 48 h with CPT-CEF increased the activity of caspase-3 in a dose-dependent manner when compared to control group. The level of caspase-3 induction in CPT-CEF treated HT29 cell lines was 30% higher at IC_50_ concentration compared to untreated cells.

### G1 phase cell cycle arrest in CPT-CEF-treated HT29 and A549 cancer cells

**Figure 12:**
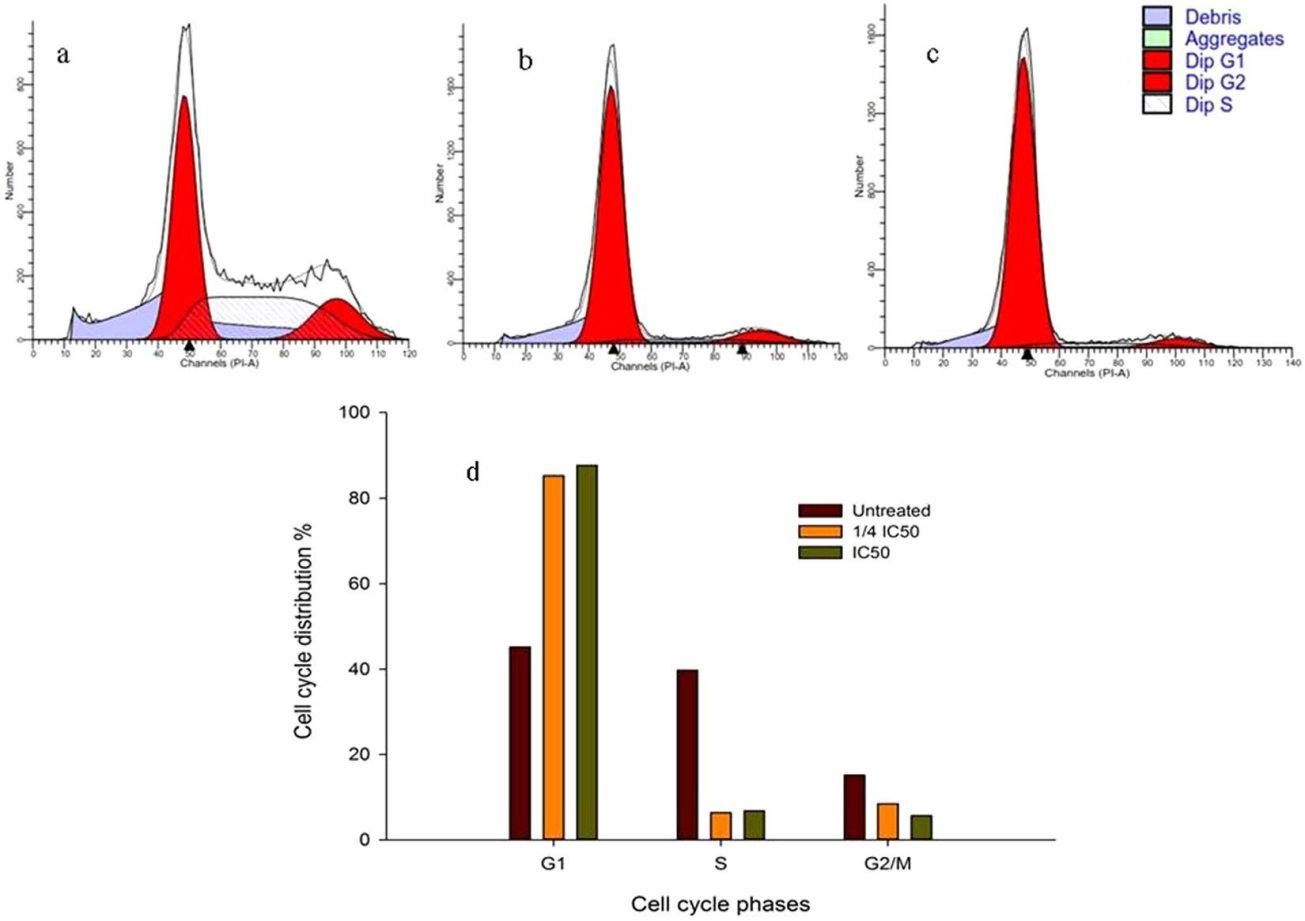
The cell cycle arrest caused by CPT-CEF in a concentration-dependent manner in HT29 cells. (**a**) Untreated, (**b**) 1/2 IC_50_ treated cells and (**c**) IC_50_ treated HT29 cells. (**d**) The graphical representation of the trend in cell cycle arrest of HT29 cells in response to CPT-CEF treatment.

**Figure 13:**
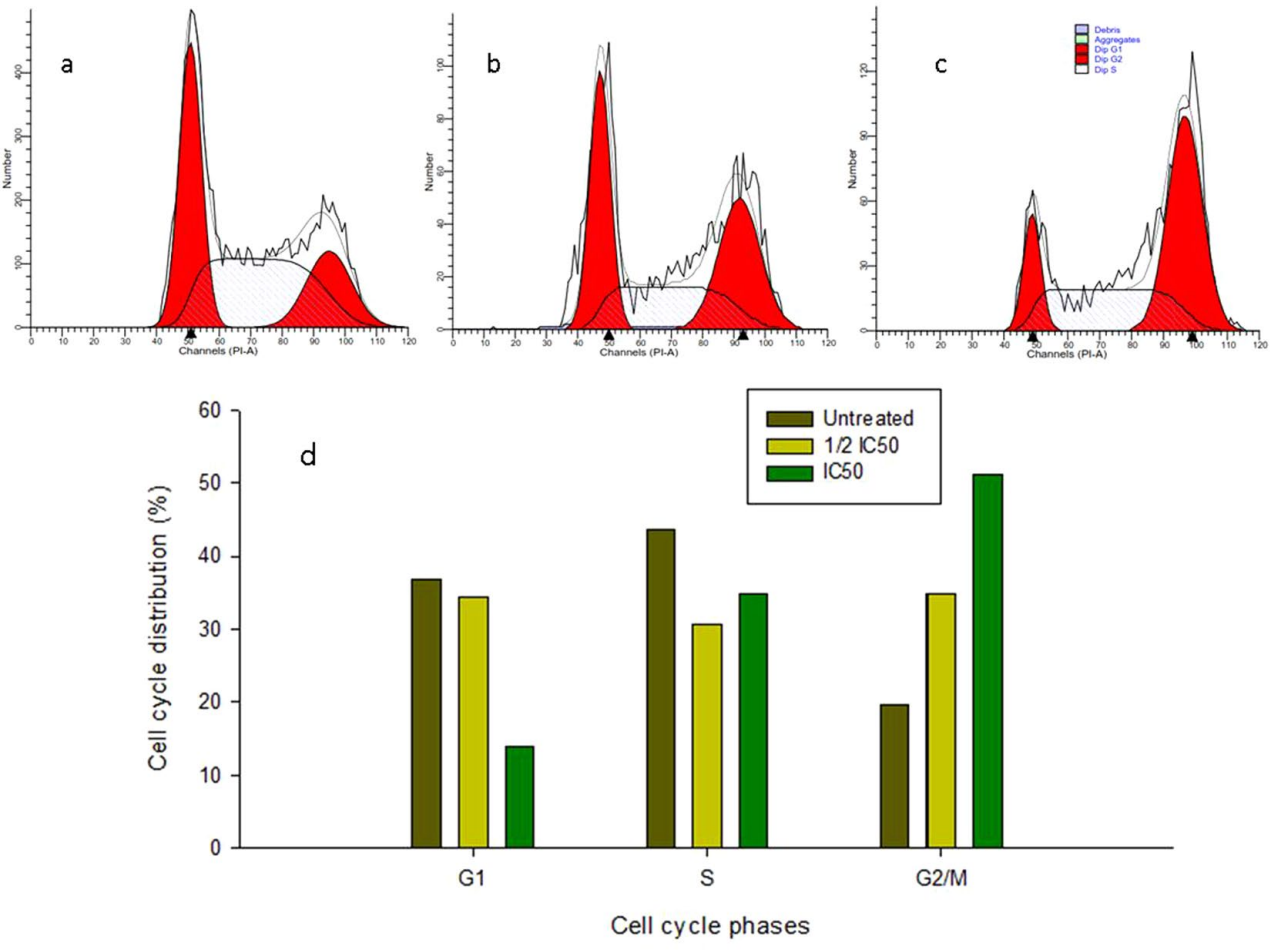
The cell cycle arrest caused by CPT-CEF in a concentration-dependent manner in A549 cells. (**a**) Untreated, (**b**) 1/2 IC_50_ treated cells and (**c**) IC_50_ treated A549 cells. (**d**) The graphical representation of the trend in cell cycle arrest of A549 cells in response to CPT-CEF treatment.

Inhibition of cell cycle progression with HT29 cells treated with CPT-CEF was evaluated at different concentrations of CPT-CEF. CPT-CEF treatment at increasing concentration showed an increasing trend in the number of HT29 cells at G1 phase and a decreasing trend in those at S phase and G2/M phase (Fig. 12). CPT is commonly known to induce cell cycle arrest at G2/M phase^23^, and, interestingly, in this study, the results show a different trend. We noticed a significant increase of cell cycle arrest in the G1 phase with a decreasing cell count in the S and G2/M phases at lower concentration of CPT-CEF. The impact of different concentrations of CPT on the stages of cell cycle arrest has been discussed previously^24^ in which low concentrations of CPT were found to cause cell cycle arrest at G2/M, while higher concentrations of CPT shifted the arrest to S phase. Interestingly, evaluation on A549 cell line showed an increasing trend in the number of cells in the G2/M phase, and a decreasing trend in the number of cells in the G1 phase, with increasing concentrations of CPT-CEF (Fig. 13) thus reflecting the nature of CPT to arrest cell cycle at G2/M phase^23^. This dissimilar data between cell lines suggests the assertion that CPT-CEF’s impact varies between different cancer cell lines.

### Magnetic force induced cells morphology changes in CPT-CEF treated HT29 cells

**Figure 14:**
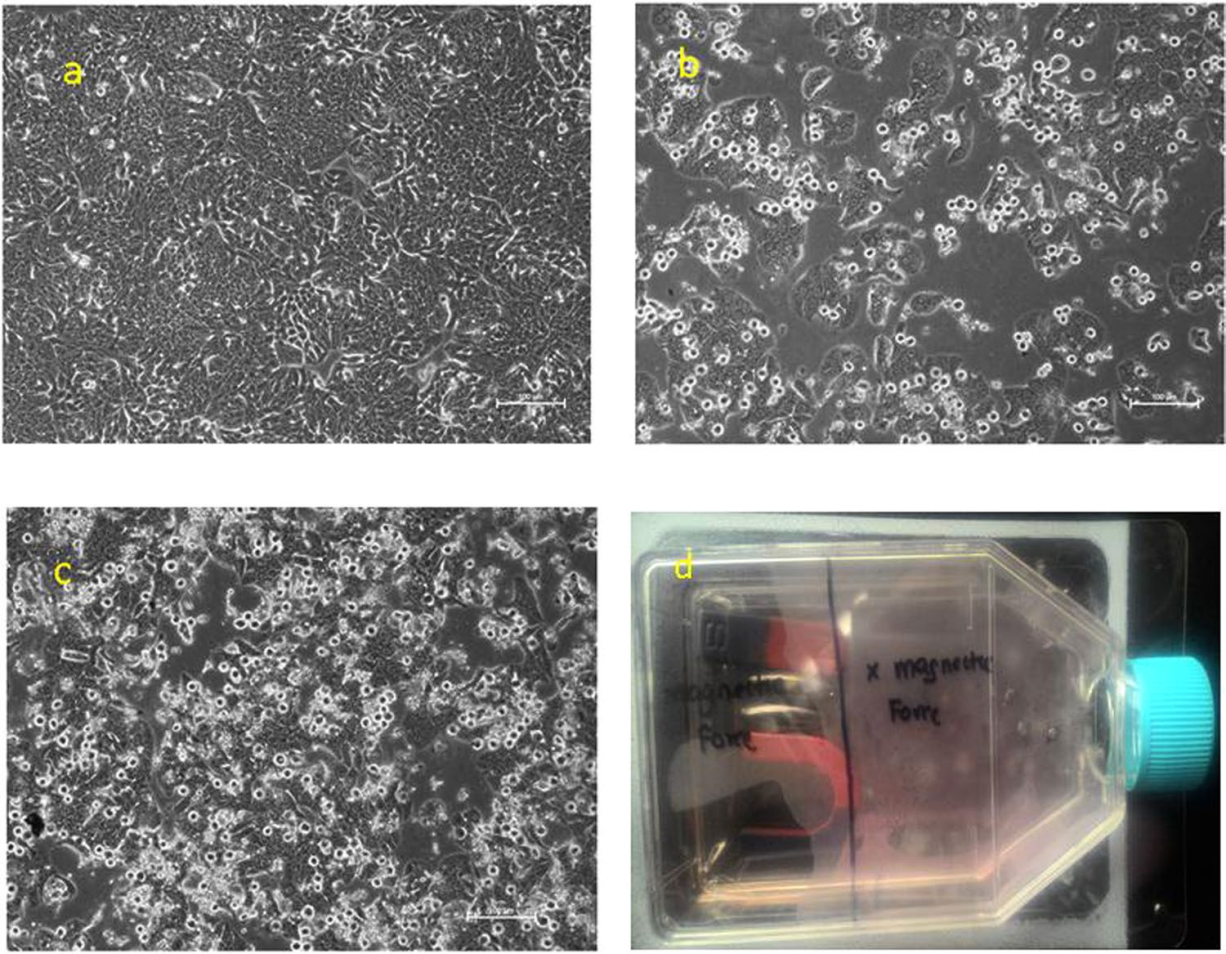
Microscopy of HT29 cells treated with CPT-CEF with magnets. Cells were exposed to the IC_50_ concentration of CPT-CEF and viewed at 48 h. Panel a (untreated HT29 cells at 48 h), (**b**) (IC_50_ treated HT29 cells for 48 h with magnets), (**c**) (IC_50_ treated HT29 cells for 48 h without magnets), (**d**) (Treated HT29-T75 flask with magnets placed under one side of the flask and another side of the flask without magnets). Under phase contrast microscopy, cells treated under magnetic environment shows significant changes in cell to cell attachment in comparison to treated cells without the influence of magnets. Microscope magnification × 100 and scale bar of 100 µm were applied for all the images.

To determine the influence of magnetic force on the CPT-CEF in cancer treatment, a simple assay utilizing magnets was performed. Figure 14a shows untreated HT29 cells without any treatment for the duration of magnetic assay. Figure 14b shows HT29 cells treated with CPT-CEF under the influence of magnets in which cell to cell attachment and cell confluency has drastically reduced in comparison to the treated side without the influence of magnets (Fig. 14c) and untreated cells (Fig. 14a). The side exposed to the magnets shows significant reduction in cell confluency and cell to cell attachment. Essentiality the area under the magnetic force is subjected towards more impact from CPT-CEF as CPT-CEF is magnetically responsive. Figure 14 d shows the treated HT29-T75 flask with magnets placed under one side of the flask and another side of the flask without magnets

## Discussion

Colon cancer is a major cancer worldwide, and despite advancements in chemotherapy, its mortality remains high, partially due to the failure of chemotherapy^25^. Camptothecin (CPT) is a major chemotherapy drug that is largely utilised in colon cancer treatment. However, the stability and solubility of CPT has greatly diminished its anti-cancer value, thus encouraging the nanotechnology field to synthesise an effective nanocarrier to enhance its solubility and stability. CPT-CEF was designed with these objectives. The formation of Fe MNs was confirmed by FT-IR spectroscopy^26^, and the particle size was found to average 168 nm. The particle size of the Fe MNs was well corroborated with TEM morphological analysis as a spherical and smooth surface. The homogenous dispersion of iron magnetic NPs by co-precipitation method was observed in a previous report^26^. After the conjugation of Fe on the EDTA-linked β-CD, the size of the carrier decreased from the non-conjugated β-CD-EDTA composite. Encapsulation of the CPT drug on the carrier subsequently increased the particle size. Conjugated Fe MNs enhance biomedical applications, such as targeted drug release and MRI detection. Because Fe MNs functionalisation imparts magnetic properties, these properties increase its usefulness with external magnetic force treatment^27, 28^. Development of magnetic nanocarriers for drug delivery applications has become a significant area of research. Fe MNs-functionalised NPs have been synthesised and their stability confirmed by poly-dispersity index. The size, charge, and surface charge of the magnetic carriers are especially critical and unequivocally influence both the blood circulation time and bioavailability of the particles inside the body^29^. Nanocarriers conjugated with Fe MNs were tested on HT29 colon cancer cells after encapsulation with CPT drug. Additionally, the magnetic studies data suggests that CPT-CEF has the potential to be utilized in targeted therapy, thus diminishing the adverse impact on other normal cells in the patient body.

In the present study, we characterised the apoptotic pathway induced by water soluble CPT-CEF in HT29 cells, which shares many features of CPT-induced apoptosis, such as nuclear condensation, and cell shrinkage. We also identified apoptosis signals, such as increased caspase-like protease activity and mitochondrial membrane depolarisation. One milligram of CPT-CEF consists of only 4.35% CPT, placing the final IC_50_ derived from the HT29 MTT assay in this study at an incredibly low range of 5.8 µg/mL of CPT per 133.5 µg/mL of CPT-CEF. At the IC_50_ of CPT-CEF, HT29 cells show a reduction of cell viability of up to 50%. In comparison, cells treated with CPT alone still maintained a cell viability of 70%. The solubility of CPT, which was enhanced through the CEF formulation, could be a contributing factor in the greater availability of CPT for the cancer cells. In addition, with only minimum amount of CPT loaded in the CEF nanocarrier, during chemotherapy only a small percentage of CPT would be administered to patients, ultimately reducing their adverse effects. Additionally, significant cell viability reduction was observed in CPT-CEF treated A549 cells. This data is indicative that CPT-CEF has the potential to be used as an anti-cancer drug in other major cancers treatment. Interestingly, we also noted that, at IC_50,_ CEF alone produced cytotoxicity. This might be due to the hemolytic effects of cyclodextrins (CDs), including β-Cyclodextrin that have been reported in several *in vitro* studies. However, the toxicological implications *in vivo* are considered negligible^30^. Essentially, the hemolytic activity of CDs correlates with their ability to solubilise cellular membrane lipids. This is due to the positive correlation that occurs between the hemolytic activity of CDs and their capacity to solubilise cholesterol, a main component of lipid bilayers, regardless of their varying physicochemical properties^31^. Additionally, the magnetic assay indicates that CPT-CEF is magnetically responsive. This is an added advantage as CPT-CEF can be used in magnetic based chemotherapy thus limiting the adverse impact caused by conventional chemotherapy.

Cell damage due to treatment with CPT-CEF indicates the ability of CPT-CEF to causes membrane damage and nuclear disintegration in cancer cells. Apoptosis is fundamental to cancer therapies such as chemotherapy. Apoptosis is characterised by particular morphological as well as biochemical alterations, such as cell shrinkage, nuclear condensation and fragmentation, plasma membrane blebbing, formation of apoptotic bodies, and loss of cell contacts with neighbouring cells^32^. Notable biochemical changes in apoptosis are chromosomal DNA cleavage into internucleosomal fragments, phosphatidylserine externalisation, and a number of intracellular substrate cleavages by specific proteases^33^. Apoptosis induction by CPT-CEF through phosphatidylserine (PS) externalisation was measured using annexin V-FITC and propidium iodide double staining. Phosphatidylserine (PS) externalisation is an early indicator of apoptosis^34^. In this study, Annexin V/PI flow cytometry assays were performed to demonstrate the apoptosis-inducing potential of CPT-CEF against HT29 cells. FITC-annexin V is commonly applied in conjugation with propidium iodide to determine early apoptosis, prior to the loss of cell membrane integrity^35^. Translocation of phosphatidylserine (PS) from the inner to the outer leaflet of the plasma membrane, or externalisation of PS to the cell surface occurs in apoptotic cells^36^. In this study, a flow cytometric FITC-annexin V/PI apoptosis detection method was used to measure the apoptosis-inducing potential of CPT-CEF. The assay demonstrated a concentration-dependent shift of healthy cells from the early to the late stage of apoptosis. It is evident from the results that CPT-CEF-induced phosphatidylserine externalisation took place by 48 h of treatment. These results further authenticated that CPT-CEF could inhibit the growth of HT29 and A549 cells by inducing apoptosis.

Mitochondria play a central role in the regulation of apoptotic signalling^37^. CPT-induced oxidative stress causes depolarisation of Δ*Ψ*_M,_ and it is associated with cellular events leading to apoptosis^38^. CPT-CEF-treated HT29 and A549 cells demonstrated a concentration-dependent decrease in the Δ*Ψ*_M,_ thus indicating that it might be an important pathway involved in the apoptotic progression of CPT-CEF induced cell death. The dissipation of Δ*Ψ*_M_ is a distinctive feature of apoptosis^39^. Mitochondrial depolarisation triggers the release of cytochrome c into the cytosol, which leads to the activation of proteases, especially the caspase cascade^20^. Caspases are key effectors of the apoptotic execution phase. Our results provide the first insight into the mechanistic pathway of apoptosis in HT29 cells induced by the novel CPT-CEF nano-compound, whereby mitochondria and caspase-like proteases play a central role.

The cell cycle is a complex process where cells receive different growth-controlling signals that are integrated and processed at various points known as checkpoints. In this study, cell cycle arrest was detected at G1 phase. Interestingly, the impact of CPT is generally attributed to G2/M phase arrest, and the application of our formulation has altered the mechanism of cell cycle arrest. This could be due to the synergistic impact contributed by the nanocarrier formulation thus altering site of cell arrest. Concurrently, treatment on the A549 cells, CPT-CEF formulation sustained the typical cell cycle arrest mechanism of CPT at G2/M phase. Taken together, these results is suggestive that the action of CPT-CEF differs according the cancer cells. Collectively, the apoptotic signals, loss of membrane potential, and nuclear degradation due to treatment with CPT-CEF in this study suggest that our CEF nanocarrier has potential to maintain the anti-cancer properties of CPT while increasing water solubility. Consistent with these results, CPT-CEF has a promising future to be equally effective in treating other major cancer in addition to colon cancer.

## Methods

### Materials

Ferrous sulphate hepta-hydrate (FeSO_4_.7H_2_O), Ferric chloride hexa-hydrate (FeCl_3_.6H_2_O), Liquid ammonia, β-Cyclodextrin, Ethylenediaminetetraacetic acid (EDTA), Disodium hydrogen phosphate (Na_2_HPO_4_.H_2_O), Polyethylene glycol (PEG), and Camptothecin (CPT) were purchased from Sigma Aldrich, Mumbai, India. Solvents such as Ethanol (C_2_H_5_OH) were purchased from Himedia Laboratories India. All chemicals were of analytical grade and were used directly as purchased without further purification. Double distilled water was used throughout the experiments.

### Synthesis of MNPs

Magnetite NPs were prepared by a previously reported method based on the controlled chemical co-precipitation^40^ of Fe^2+^ and Fe^3+^ (1:2 ratio) in an ammoniacal medium at 80 °C under a nitrogen atmosphere. In a typical synthesis, 0.02 M of ferrous sulphate (FeSO_4_·7H_2_O) and 0.04 M of FeCl_3_·6H_2_O were dissolved in 200 mL of deionised water. The mixture was stirred and heated to 80 °C under a nitrogen atmosphere; 12 mL of a 25% ammonia solution was injected into the flask. Stirring was continued for 20 min to allow the growth of the NPs. After 20 min, the solution was cooled to 28 °C and the resulting magnetites NPs were centrifuged. The NPs were washed three times with distilled water. The pH of the suspension was brought to neutral by adding dilute HCl, and the particles were rewashed with distilled water for further experiments.

### Synthesis of EDTA-β-CD

EDTA-β-CD polymers were synthesised by reacting β-CD with EDTA as a cross-linker and disodium hydrogen phosphate (MSP) as a catalyst. β-CD (4 g, 3.5 mmol), EDTA (6 g, 20.4 mmol), MSP (Na_2_HPO_4_ 7H_2_O, 2.68 g, 10 mmol), and 20 mL of deionised water were mixed in a round-bottomed flask and stirred in a 100 °C oil bath for 1 h. Polyethylene glycol 200 (PEG-200, 0.5 g, 2.5 mmol) as a dispersant was added drop wise to help to dissolve β-CD in the water. The mixture was transferred into a petri dish (ϕ160 mm) and heated in an oven at 155 °C for 10 h. After cooling to room temperature, the resulting condensation polymer product was ground and soaked with 500 mL of deionised water, and then suction-filtered and rinsed with a large amount of 0.1 M HCl, deionised water, 0.1 M NaOH, deionised water, and methanol, to remove the unreacted materials and catalyst. The final product was dried under vacuum at 60 °C overnight^41^.

### Synthesis of β-CD-EDTA with Fe_3_O_4_

Initially, 0.03 g of β-CD-EDTA was dissolved in 10 mL of double distilled water; 0.03 g of MNPs was dissolved in 1.5 mL of PBS 6.8 solution. The MNPs containing solution was added to the β-CD-EDTA solution with continuous stirring at 1,000 rpm for 3 hours. The NP solution was then centrifuged at 4,000 rpm for 15 min. Finally, the NPs were dried at 37 °C for 24 h.

### Preparation of drug loaded MNPs

The β-CD-EDTA modified with Fe_3_O_4_ NPs encapsulated with camptothecin was prepared in a phosphate buffer solution (PBS)/ethanol system using a co-lyophilisation technique. Twenty micrograms of β-CD-EDTA modified with Fe_3_O_4_ and 1.0 mg of CPT were dissolved in the mixed solvent system, i.e., 2 mL of PBS and 2 mL of ethanol. The system was left to equilibrate under constant stirring for 24 h at 50 ° C in the dark. After the organic solvent was completely evaporated under vacuum, the suspension was filtered. The filtrate containing β-CD-EDTA modified with Fe_3_O_4_ (β-CD-EDTA-Fe_3_O_4_) was lyophilised to obtain a dry yellow powder^42^.

### Characterisation studies

#### FT-IR analysis

A small quantity of Fe_3_O_4_, drug-unloaded nanocarrier and drug-loaded nanocarrier was separately mixed with 200 mg KBr and compressed to form tablets. These tablets were scanned on a Fourier Transform Infrared Spectrometer (Spectrum GX-1, PerkinElmer, USA), in the spectral region of 4,000–400 cm^−1^.

#### Transmission Electron Microscopy (TEM)

The structural morphology and crystallite size of the samples were further investigated via high resolution transmission electron microscopy (HRTEM, TECNAI F30). For HRTEM analysis, the as-synthesised NP and its composites were dispersed in ethanol with the help of ultrasonication for 15 min and then loaded on a carbon-coated copper mesh.

#### Particle size analysis

Mean particle size (diameter, nm ± S.D.) and polydispersity index of the NPs were determined using BECKMAN COULTER, Delsa^TM^ Nano C. Measurements were at a 90° angle at 25 °C under suitable dilution conditions, and were performed in triplicate.

#### Zeta potential measurement

Zeta potential of NP dispersions was measured in mV by BECKMAN COULTER, Delsa ^TM^ Nano C in triplicate to determine the surface charge and the potential physical stability of the nanosystem. Zeta potential of NPs was measured in aqueous dispersion. Measurements were at a 120° angle at 25 °C, and were performed in triplicate.

#### *In-vitro* drug release profile

*In-vitro* release profiles of CPT from CEF nanocarrier were examined for 100 minutes in acidic medium (pH 2.4) and PBS solution (pH 7.0). Dialysis technique was employed. The nanoparticles (10 mg) were placed in a dialysis tube with 5 mL of release medium (MWCO: 12,000 Da). The dialysis tube was then placed in 50 mL of double distilled water at 37 °C and stirred continuously at 500 rpm. At specific time intervals, 2 mL of solution was withdrawn from the outer compartment and replaced with fresh double distilled water (2 mL). The concentration of the released CPT was determined by UV spectrophotometer at λmax 260 nm. The analysis was performed in triplicate for each sample.

#### Magnetic moment analysis

Magnetic properties of the iron nanoparticles and iron nanoparticles loaded nanocarriers (CEF) are tested with two different analyses. The magnetic moment of the samples was determined in vibrating sample magnetometer (VSM, Dexing, Model: 250) with a sensitivity of 50 emu.

## Biological application studies

### Materials

Annexin V-FITC apoptosis detection kit and mitochondrial depolarisation membrane kit were purchased from BD Bioscience (USA). 3-(4,5-Dimethylthiazol-2-yl)-2,5-diphenyltetrazolium bromide (MTT), Roswell Park Memorial Institute (RPMI) 1640 Medium, Foetal bovine serum (FBS), Penicillin (100 U/mL)/Streptomycin (100 µg/mL), 0.25% Trypsin-EDTA were acquired from Naccalai, (Japan). The caspase-3 colorimetric assay kit was obtained from R&D systems Co. (Minneapolis, USA), and the cell cycle analysis kit was obtained from Abcam (USA).

### Cell culture

HT29: Human colorectal adenocarcinoma cells and A549: Adenocarcinomic human alveolar basal epithelial cells were procured from the Laboratory of Vaccine and Immunotherapy (LIVES) Institute of Biosciences (IBS), UPM. The cell lines were grown adherently using RPMI media supplemented with 10% foetal calf serum, 100 U/mL penicillin, and 100 μg/mL streptomycin at 5% CO_2_ at 37 °C. The cells were seeded in 96-well plates at a concentration of 1 × 10^4^ in 100 µl of cell culture medium. Cells were allowed to grow for 24 h to reach approximately 90% confluency.

### Treatment with CPT-CEF

The final stock solution of each compound was made by dissolving them in 10% DMSO and cell culture media. Multiple concentrations were made by serial dilutions, using cell culture medium. DMSO concentration was kept below 1% (v/v) in all analyses. As vehicle control, complete cell culture medium was added to the cells without imposing any treatment.

### MTT viability assay

The tetrazolium salt 3-[4, 5-demethylthiazol-2-yl]-2-5-diphenlytetrazolium bromide (MTT) assay was performed to determine cell viability and overall cytotoxicity with different concentrations of CPT-CEF on HT29 (human colon adenocarcinoma) and A549 (human lung adenocarcinoma). Cell viability of cancer cell lines in response to treatment with various concentrations of CEF-CET, CPT, CEF, and FMN were determined using MTT as described by Mosmann^43, 44^. Cells were plated at a density of 1 × 10^4^ cells per well in 96-well plates and cultured at 37 °C for 24 h under 5% CO_2_ for cell attachment. Cells were then treated with the compounds mentioned above. The concentrations used were 250, 125, 62.5, 31.25, 15.62, 7.81, 3.91, and 1.95 µg/mL. The final concentration of DMSO in the well did not exceed 1% (v/v). Treated cells were then tested after 24, 48, and 72 h of incubation. For each dosage, three replicates were performed. Negative controls were performed with cell culture media only. The MTT assay was then performed. The following procedure was conducted in dim light, as MTT is light-sensitive. To initiate the assay, 20 µl of MTT (Naccalai, Japan) (5 mg/mL) was added into each well and incubated for 3 h at 37 °C. After incubation, supernatants were carefully removed and 100 µl of DMSO were then added into each well to solubilise the formazan product. The absorbance was measured using a plate reader (Sunrise™-Tecan) at 570 nm with a reference wavelength of 630 nm. Cell viability was calculated as the ratio of the absorbance of treated cells to that of blank controls. The IC_50_ value of CPT-CEF was determined, and this concentration was utilised for subsequent assays.

### Annexin V/ PI Assay

Detection of apoptosis was conducted using the Annexin VFITC/PI apoptosis detection kit (BD Pharmingen™, USA), according to manufacturer’s protocol. Briefly, cells were plated at a density of 3 × 10^5^ per well in six-well plates, and treated with different concentrations of CPT-CEF. After a 48-h incubation period, cells were collected, pooled, and washed with PBS twice. Cells were then resuspended in 1X Binding Buffer at a concentration of 1 × 10^6^ cells/mL, and 100 µL of the solution (1 × 10^5^ cells) was transferred to a 5 mL culture tube; 5 µL of FITC Annexin V and 5 µL of PI was then added into the tube and incubated for 15 min at room temperature in the dark. Then, 400 µL of 1X Binding Buffer was again added into each tube, and the contents were examined using a BD FACSARIA flow cytometer (USA).

### Mitochondrial depolarisation assay (JC-1)

Mitochondrial depolarisation was determined using a JC-1 kit ((BD Pharmingen™, USA). The cell treatment procedure was as described in the Annexin V/PI assay treatment. Following treatment, 1 mL of each cell suspension was transferred into a sterile 15 mL polystyrene centrifuge tube. Cells were then centrifuged at 400 × g for 5 min at room temperature, and the supernatant was discarded; 0.5 mL of freshly prepared JC-1 Working Solution was added into the tubes and incubated for 10–15 min at 37 °C in a CO_2_ incubator. Cells were then washed twice with 1 × Assay Buffer and centrifuged 400 × g for 5 min. Cells were finally resuspended in 0.5 mL 1 × Assay Buffer and analysed using flow cytometry.

### Cell cycle analysis

Cells were treated and incubated for 48 h, as explained above. Cell cycle analysis was carried out using a Propidium Iodide Flow Cytometry Kit for Cell Cycle Analysis (Abcam, UK). Cells (3 × 10^5^ per well) were grown in six-well plates and then treated with multiple concentrations of CPT-CEF for 48 h. After treatment, the culture media was removed, and cells were rinsed with PBS. Trypsin was used to dissociate the cells. Culture media and PBS rinses were collected and pooled. Cells were then pelleted by centrifugation at 500 × g for 5 min. The supernatant was then discarded, and the cells were washed with 1X PBS and centrifuged again at 500 × g for 5 min. Cells were then fixed using 66% ethanol on ice, and stored at 4 °C for at least 2 h. The cells were then centrifuged at 500 × g for 5 min and washed with 1 mL 1X PBS and centrifuged again. The cells were gently resuspended in 200 µL of 1X propidium iodide + RNAse staining solution. After incubation for 30 min in the dark at 37 °C, cells were analysed for DNA content by using a FACS calibur flow cytometer. Cell distribution among cell cycle phases and the percentage of apoptotic cells were evaluated as previously described^45^. The cell cycle distribution is shown as the percentage of cells containing 2n (G1 phase), 4n (G2 and M phases), and 4n > 3 > 2n DNA amount (S phase), assessed via PI staining. The apoptotic population is defined by the percentage of cells with DNA content lower than 2n (sub/G1 phase).

### Caspase-3

Cells (1 × 10^5^ per well) were cultured overnight in six-well plates, and then treated with various concentrations of CPT-CEF (30, 60, and 130 µg/mL) for 48 h. Cells without treatment were used as controls. Caspase-3 activity was assessed, according to the manufacturer’s instruction of the caspase-3 colorimetric Assay Kit (R&D systems, USA). Briefly, cells were harvested after treatment and lysed in 50 µL lysis buffer on ice for 10 min and then centrifuged at 10,000 × g for 1 min. After centrifugation, 50 µl of supernatant were incubated with caspase-3 substrate in reaction buffer. Samples were incubated in 96-well, flat bottom microplates at 37 °C for 2 h. The amount of released pNA (p-nitroaniline) was measured using a microplate reader (Bio-Rad, Hercules, CA, USA) at 405 nm wavelength. Background readings were determined from wells containing culture medium without cells and without substrate. Protein concentration was determined using the Pierce 660 nm Protein Assay Reagent.

### AO/PI Staining

The morphological changes in CPT-CEF treated HT29 and A549 cells were characterised using acridine orange (AO) and propidium iodide (PI) double staining, according to the method described by Hajiaghaalipour *et al*.^46, 47^ with minor modifications. Briefly, cells were plated at a density of 1 × 10^5^ cells/mL in a six-well plate and treated with the IC_50_ concentration of CPT-CEF in an atmosphere of 5% CO_2_ at 37 °C for 48 h. The cells were then trypsinized with trypsin-EDTA, washed twice with PBS and centrifuged for 5 min to remove the remaining media. An equal volume of fluorescent dye (AO/PI) containing AO (50 μg/mL) and PI (50 μg/mL) was added to the cellular pellet, and freshly stained cells were observed under a UV-fluorescence microscope within 30 min.

### Magnetic assay

A simple magnetic assay was performed to indicate the magnetic potential of CPT-CEF. To perform this, a T75 flask confluent with HT29 cells was treated with the IC_50_ concentration of CPT-CEF. This treated flask was then placed on layer magnets that are directed towards one side of the flask while the other side is left free without any magnets. Post 48 h of treatment, changes in the cell morphology was observed and compared with the sides of the flask with magnets and side of the flask without magnets.

### Statistical analysis

Results were expressed as the mean ± standard deviation (SD). Statistical comparisons of mean values were analysed by one-way ANOVA using SPSS 22.0 software. All P < 0.05 was considered to indicate statistically significant differences.

## Conclusion

In this study, we have thoroughly studied the mechanism of action of CPT-CEF by analysing the nuclear, mitochondrial membrane potential, activity of caspase-like proteases, and cytosolic changes associated with apoptosis in HT29 cells. CPT-CEF induced cell apoptosis and growth inhibition due to cell cycle arrest, as well as activation of mitochondrial apoptotic pathways. In the present study, we demonstrated that the soluble form of CPT-CEF has successfully exhibited anti-cancer properties while being loaded with a low concentration of CPT as well as being magnetically active. Also, the selected assay performed in A549 cells are also reflective on the ability of CPT-CEF to be utilized in the treatment of other cancers apart from colon cancer. With further improvements, this new formulation could be a promising nanocarrier for CPT drug delivery for an effective chemotherapy treatment of colon cancer.

## Electronic supplementary material

Supplementary information
