# Supplementary material for: Efficiency of newly formulated camptothecin with β-cyclodextrin-EDTA-Fe3O4 nanoparticle-conjugated nanocarriers as an anti-colon cancer (HT29) drug
Source: Sci Rep. 2017 Sep 8;7:10962. doi: 10.1038/s41598-017-09140-1 (PMC5591276; doi:10.1038/s41598-017-09140-1)
Supplement: Supplementary file 1 — Supplementary information [file 41598_2017_9140_MOESM1_ESM.doc]

**Efficiency of newly formulated camptothecin with β-cyclodextrin-EDTA-Fe_3_O_4_ nanoparticle-conjugated nanocarriers as an anti-colon cancer (HT29) drug**

**Running title: Efficiency of camptothecin with β-cyclodextrin-EDTA-Fe_3_O_4_ nanoparticle-conjugated nanocarriers as an anti-colon cancer (HT29) drug**

Poorani Krishnan,^1^ Mariappan Rajan,^2*^ Sharmilah Kumari,^1^ S. Sakinah,^1^ Sivan Padma Priya,^1^ Fatin Amira,^1^ Lawal Danjuma,^1^ Mok Pooi Ling,^3,4^ Sharida Fakurazi,^5^ Palanisamy Arulselvan,^5 ,12^ Akon Higuchi,^6,7,8^ Ramitha Arumugam,^9^ Abdullah A. Alarfaj,^8^ Murugan A. Munusamy,^8^ Rukman Awang Hamat,^1^ Giovanni Benelli,^10^ Kadarkarai Murugan,^11^ and S. Suresh Kumar^1,3,*^

^1^Department of Medical Microbiology and Parasitology, Universiti Putra Malaysia, 43400 UPM Serdang Selangor, Malaysia.

^2^Department of Natural Products Chemistry, School of Chemistry, Madurai Kamaraj University, Madurai 625 021, Tamil Nadu, India

^3^Department of Biomedical Science, Universiti Putra Malaysia, 43400 UPM Serdang Selangor, Malaysia.

^4^Genetics and Regenerative Medicine Research Centre, Universiti Putra Malaysia, 43400 UPM Serdang, Selangor, Malaysia

^5^Laboratory of Vaccines and Immunotherapeutic, Institute of Bioscience, Universiti Putra Malaysia, 43400 UPM Serdang Selangor, Malaysia.

^6^Department of Chemical and Materials Engineering, National Central University, Jhong-li, Taoyuan, 32001 Taiwan

^7^Department of Reproduction, National Research Institute for Child Health and Development, Tokyo 157-8535, Japan

^8^Department of Botany and Microbiology, King Saud University, Riyadh 11451, Saudi Arabia

^9^Department of Biology, Faculty of Science, Universiti Putra Malaysia, 43400 UPM Serdang Selangor, Malaysia.

^10^Department of Agriculture, Food and Environment, University of Pisa, via delBorghetto 80,56124 Pisa, Italy.

^11^Division of Entomology, Department of Zoology, School of Life Sciences, Bharathiar University, Coimbatore, Tamil Nadu, India.

^12^Muthayammal Centre for Advanced Research, Muthayammal College of Arts and Science, Rasipuram, Namakkal, Tamilnadu, 637408, India.

* Author to Whom Correspondence should be addressed:

Dr. Suresh Kumar E-mail: [sureshkudsc@gmail.com](mailto:sureshkudsc@gmail.com); Tel: +60389472371; +60146337312


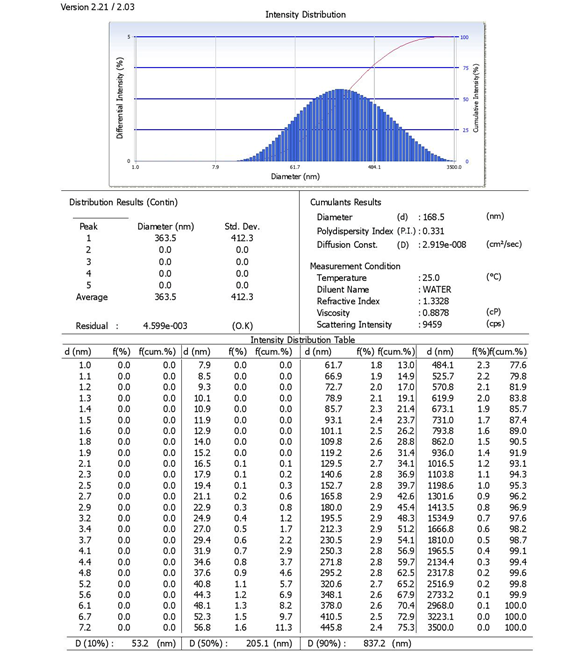
**Supplementary figures**

Figure S1. Particle size and Poly Dispersity index analyses of Fe_3_O_4_.

**
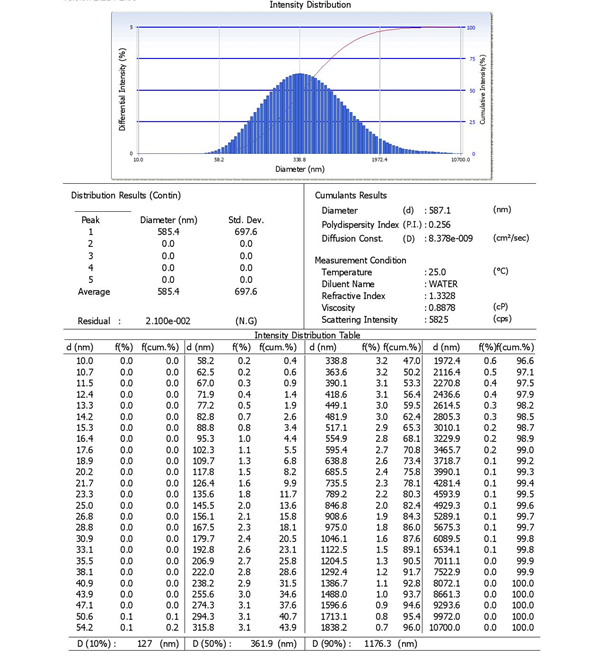
**

Figure S2. Particle size and Poly Dispersity index analyses of β-CD-EDTA.


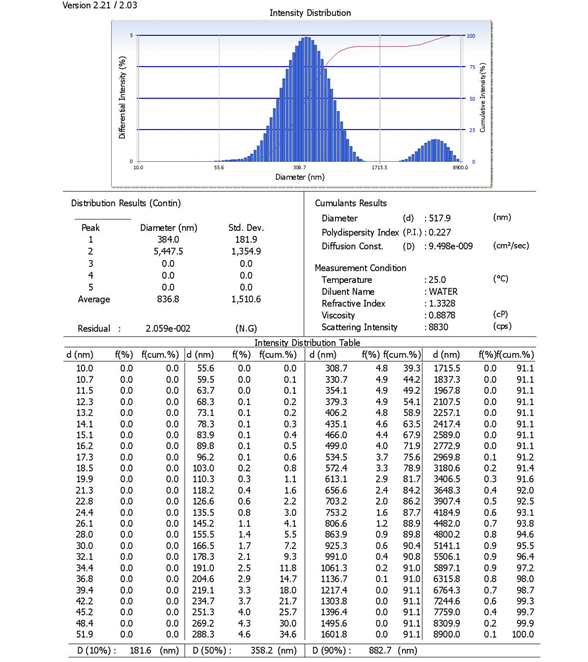


Figure S3. Particle size and Poly Dispersity index analyses of β-CD-EDTA-Fe_3_O_4_.


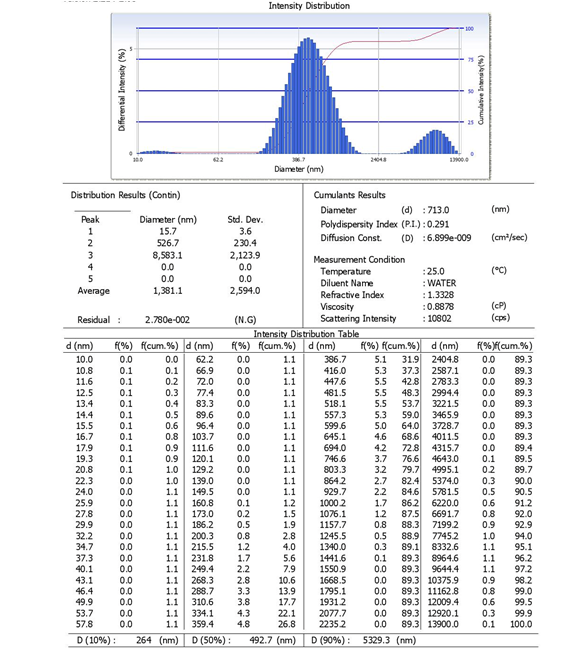


Figure S4. Particle size and Poly Dispersity index analyses of β-CD-EDTA-Fe_3_O_4_/CPT.


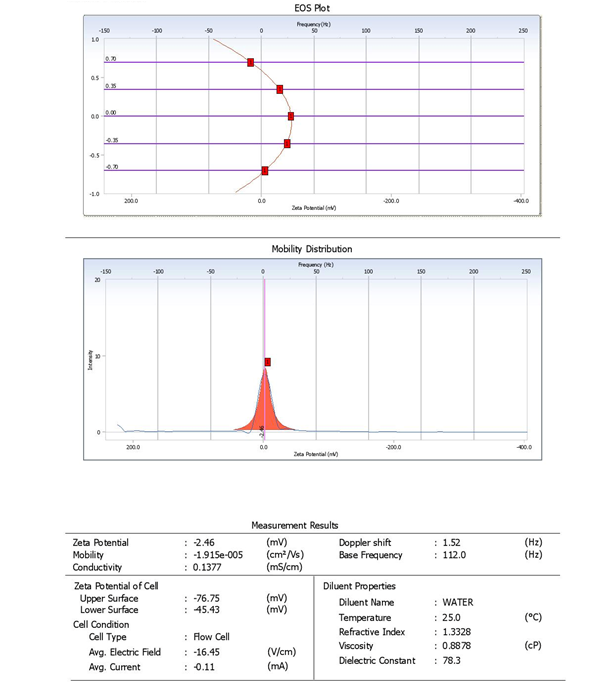


Figure S5. Zeta potential analysis of β-CD-EDTA-Fe_3_O_4_


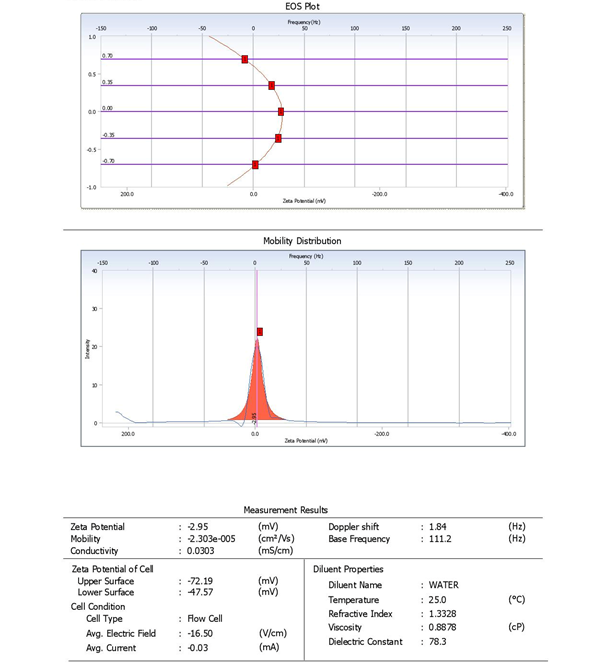


Figure S6. Zeta potential analyses of β-CD-EDTA-Fe_3_O_4_/CPT
